# Room-temperature serial synchrotron crystallography of the human phosphatase PTP1B

**DOI:** 10.1107/S2053230X22011645

**Published:** 2023-01-01

**Authors:** Shivani Sharma, Ali Ebrahim, Daniel A. Keedy

**Affiliations:** a Structural Biology Initiative, CUNY Advanced Science Research Center, New York, NY 10031, USA; b PhD Program in Biology, CUNY Graduate Center, New York, NY 10016, USA; c Department of Chemistry and Biochemistry, City College of New York, New York, NY 10031, USA; d PhD Programs in Biochemistry, Biology and Chemistry, CUNY Graduate Center, New York, NY 10016, USA; King’s College London, United Kingdom; University of Padova, Italy

**Keywords:** X-ray crystallography, phosphatases, apo protein tyrosine phosphatase 1B, allostery, room-temperature serial crystallography

## Abstract

Room-temperature X-ray crystallography provides unique insights into protein conformational heterogeneity, but obtaining sufficiently large protein crystals is a common hurdle. Serial synchrotron crystallography (SSX) helps to address this hurdle by allowing the use of many medium- to small-sized crystals. Here, a recently introduced serial sample-support chip system has been used to obtain the first SSX structure of a human phosphatase, specifically protein tyrosine phosphatase 1B (PTP1B) in the unliganded (apo) state. In previous apo room-temperature structures, the active site and allosteric sites adopted alternate conformations, including open and closed conformations of the active-site WPD loop and of a distal allosteric site. By contrast, in our SSX structure the active site is best fitted with a single conformation, but the distal allosteric site is best fitted with alternate conformations. This observation argues for additional nuance in interpreting the nature of allosteric coupling in this protein. Overall, our results illustrate the promise of serial methods for room-temperature crystallography, as well as future avant-garde crystallography experiments, for PTP1B and other proteins.

## Abstract 2

The first serial synchrotron crystallographic structure of apo PTP1B is reported and the conformational heterogeneity at several allosteric sites is compared with that seen in prior structures.

## 1. Introduction

X-ray crystallography offers detailed insights into protein structure. Although most X-ray crystallography is performed with the sample at cryogenic temperature (cryo), data collection at elevated temperatures including room temperature (RT) offers unique insights into protein conformational heterogeneity and function (Fraser *et al.*, 2009 ▸, 2011 ▸; Keedy *et al.*, 2014 ▸, 2015 ▸; Fischer *et al.*, 2015 ▸; Milano *et al.*, 2022 ▸). Previously, a barrier to RT crystallography was growing sufficiently large crystals to counteract the increased sensitivity to radiation damage at RT relative to cryo (Garman & Nave, 2009 ▸; Warkentin *et al.*, 2013 ▸). However, new methods are emerging to enable RT crystallography (Fischer, 2021 ▸). In particular, serial crystallography methods which enable the use of hundreds to thousands of microcrystals instead of larger macrocrystals have been developed for X-ray free-electron lasers (XFELs; Hirata *et al.*, 2014 ▸; Moreno-Chicano *et al.*, 2019 ▸) and have also been applied at synchrotrons in an approach known as serial synchrotron crystallography (SSX; Owen *et al.*, 2017 ▸; Diederichs & Wang, 2017 ▸).

Although radiation-damage effects cannot be mitigated to the same degree at synchrotrons compared with XFELs, SSX nevertheless permits low-dose data collection, which is advantageous over single-crystal studies when studying radiation-sensitive proteins, such as those containing disulfide bonds and transition metals (Ebrahim, Moreno-Chicano *et al.*, 2019 ▸). In addition, an advantage of SSX is the ability to average over many crystals, as was performed in the current study, to ameliorate single-crystal bias; such multi-crystal averaging is also important for other applications such as isolating bound ligands in crystallographic screens (Pearce *et al.*, 2017 ▸). In some favorable cases it is also possible to isolate different snapshots of protein conformation(s) within the same data set, for example by isolating distinct crystal polymorphs that correspond to independent structures (Ebrahim, Appleby *et al.*, 2019 ▸).

Recently, a new serial sample-support system for SSX was introduced (Illava *et al.*, 2021 ▸). In this approach, a vacuum source in a humid chamber is used to load microcrystals onto a chip, which has a base that is compatible with standard goniometers at synchrotron beamlines. The system can be used at cryo, but is particularly valuable for RT. It has previously been demonstrated for three proteins: fluoroacetate dehalogenase, hen egg-white lysozyme and human glutaminase C (Illava *et al.*, 2021 ▸; Milano *et al.*, 2022 ▸).

Here, we exploit this serial system for a distinct, biomedically relevant protein target: human protein tyrosine phosphatase 1B or PTP1B (also known as PTPN1). PTP1B is the archetypal protein tyrosine phosphatase, playing a key role in cellular regulation, cell-growth signaling and signal transduction by removing phosphate groups from phosphorylated tyrosine residues. Dysregulation of PTP1B function has been attributed to a number of human pathologies and has led to PTP1B becoming a validated therapeutic target (Zhang & Zhang, 2007 ▸) for several diseases: diabetes (Zhang & Lee, 2003 ▸; Montalibet & Kennedy, 2005 ▸), via its critical role as a negative regulator of insulin receptor signaling by dephos­phorylating the active form of insulin receptor kinase (Drake & Posner, 1998 ▸); cancer (Tonks & Muthuswamy, 2007 ▸), including a key role in the PKM2/AMPK/mTOC1 pathway, whereby inhibiting PTP1B leads to the arrest of pancreatic cancer cell proliferation (Xu *et al.*, 2019 ▸); Alzheimer’s disease, where increased PTP1B activity is associated with impaired neuronal insulin and leptin signaling (Vieira *et al.*, 2017 ▸); and Rett syndrome, where PTP1B overexpression is a specific biomarker and PTP1B inhibition leads to improvement of some Rett syndrome phenotypes (Krishnan *et al.*, 2015 ▸). Because active-site inhibitors of PTP1B suffer from bioavailability and selectivity limitations (Stanford & Bottini, 2017 ▸), notwithstanding some progress in bypassing such limitations (Zhang, 2001 ▸), there has been an increasing focus on the potential of allosteric inhibition. Several allosteric inhibitors targeting different sites in PTP1B have been reported in the literature (Wiesmann *et al.*, 2004 ▸; Hansen *et al.*, 2005 ▸; Krishnan *et al.*, 2014 ▸, 2018 ▸; Keedy *et al.*, 2018 ▸; Friedman *et al.*, 2022 ▸). However, to date none have been clinically approved, illustrating the persistent need to elucidate conformational ensembles and allosteric mechanisms in this protein.

As noted above, RT crystallography can reveal alternate protein conformations, which can interact with one another to imbue proteins with allosteric properties (van den Bedem *et al.*, 2013 ▸). For PTP1B, 295 cryo structures are available in the Protein Data Bank (PDB; Berman *et al.*, 2000 ▸), but only seven structures ranging from above the glass transition (>180 K) to RT are available, only three of which are of the apo enzyme. The first two such apo structures (PDB entries 6b8x and 6b8t) are from multitemperature crystallography of PTP1B, which indicated the existence of an extensive allosteric network spanning several promising sites in the protein (Keedy *et al.*, 2018 ▸). Although these two structures are nominally apo, the crystals included high concentrations of glycerol, resulting in glycerol molecules that bound in the active site and ostensibly biased the conformational ensemble of the protein (Keedy *et al.*, 2018 ▸). The remaining apo RT structure of PTP1B, PDB entry 7rin, has the same crystal lattice as PDB entry 6b8x, sharing a similar unit cell and crystallization conditions, although it differs slightly in resolution (PDB entry 6b8x, 1.74 Å; PDB entry 7rin, 1.85 Å) and data-collection temperature (PDB entry 6b8x, 278 K; PDB entry 7rin, 295 K). Despite being described in the PDB metadata as being collected at 277 K, the diffraction data for PDB entry 2cm2 were actually collected at 93.15 K as per the crystallographic statistics presented in the accompanying manuscript (Ala *et al.*, 2006 ▸). No serial crystallography structures of PTP1B, nor indeed, to our knowledge, of any human phosphatase, are yet available.

Here, we provide the first serial synchrotron crystallography (SSX) structure of PTP1B, demonstrating the feasibility of this method for this protein. We use two different processing pipelines and obtain very similar results with both, demonstrating the robustness of processing data from the SSX chips used here. Our data set allows us to draw comparisons to other existing structures with regard to the conformational ensemble of truly apo PTP1B. As may be expected, our model is broadly similar to the dozens of ligand-bound cryo structures and the smaller set of RT structures in different conditions. However, despite its moderate resolution relative to past single-crystal structures, our data set also indicates a degree of allosteric decoupling that adds nuance to the previously reported paradigm of allostery in this protein (Keedy *et al.*, 2018 ▸). Finally, our experiments pave the way for future serial crystallography experiments on PTP1B and related proteins.

## 2. Materials and methods

### 2.1. Protein expression and purification

PTP1B was expressed and purified as reported previously (Keedy *et al.*, 2018 ▸). In brief, we used a ‘wild-type’ PTP1B known as WT* containing the C32S/C92V double mutation, residues 1–321, in a pET-24b vector resistant to kanamycin. WT*-transformed *Escherichia coli* BL21 colonies were selected against LB + kanamycin plates and used to inoculate 5 ml starter cultures of LB + kanamycin (1 m*M* final concentration) grown overnight at 37°C with shaking. Starter cultures were subsequently used to inoculate 1 L growth cultures of LB + kanamycin (1 m*M* final concentration), induced with 100 µ*M* isopropyl β-d-1-thiogalactopyranoside and grown for a further 4 h at 37°C. Cell pellets were harvested via centrifugation and stored at −80°C until needed, or immediately sonicated (on ice) for 10 min with 10 s on/off at an amplitude of 50%.

PTP1B WT* was initially purified via cation exchange on an SP FF 16/10 HiPrep column (GE Healthcare Life Sciences) in lysis buffer (100 m*M* MES pH 6.5, 1 m*M* EDTA, 1 m*M* DTT) using a NaCl gradient (0–200 m*M*), with the protein eluting around 200 m*M* NaCl. Size-exclusion chromatography was subsequently performed on a Superdex 75 size-exclusion column (GE Healthcare Life Sciences) in crystallization buffer (10 m*M* Tris pH 7.5, 0.2 m*M* EDTA, 25 m*M* NaCl, 3 m*M* DTT). Purity was assessed by SDS–PAGE and the protein was found to be pure and contamination-free.

### 2.2. Crystallization

WT* PTP1B was used at 40 mg mL^−1^ and drops were set up in 96-well plates using an SPT Labtech Mosquito Xtal3 with a ratio of 0.1 µL protein solution and 0.1 µL well solution (0.1 *M* MgCl_2_, 0.1 *M* HEPES pH 7.0, 12–14.5% PEG 4000) and were incubated at 4°C. Crystals grew within 24 h and continued growing for a few more days, reaching final sizes of ∼50–100 µm.

### 2.3. X-ray data collection

Samples were loaded onto the MiTeGen SSX sample supports as reported by Illava *et al.* (2021 ▸) and discussed here in brief. PTP1B crystals and sample supports were placed into a humidified glove box (>97% relative humidity) in order to prevent the crystals from drying out on the support during the vacuum-loading process. Sample supports were seated within a vacuum port and 3–5 µl of PTP1B crystals in mother liquor were applied to the support surface. A light vacuum was applied to remove excess mother liquor from the support, with the support then being sealed using Mylar film of thickness 2.5 µm.

Data were collected from the PTP1B crystals at room temperature (25.5 ± 1°C) on the ID7B2 (FlexX) beamline for macromolecular X-ray science at the Cornell High Energy Synchrotron Source (MacCHESS), Ithaca, New York, USA. Crystal-loaded MiTeGen SSX sample supports were mounted on the ID7B2 endstation goniometer and rastered through the X-ray beam. The sample support was rastered in steps of 20 µm between likely crystal positions. At each such position six data frames were collected, and four blank frames were then collected during transit to the next position. The six data frames consisted of either 0.2° or 0.5° oscillations depending on the chip, with a total oscillation of either 1.2° or 3.0° collected per wedge. Initial data quality and resolution limits were assessed at ID7B2 using the *ADX* software suite. The photon flux was 10^11^ photons s^−1^, allowing the calculation of an estimated diffraction-weighted dose (DWD) per crystal of <35.1 kGy using *RADDOSE*-3*D* (Bury *et al.*, 2018 ▸), which is less than the 0.38 MGy (380 kGy) limit proposed for RT SSX (de la Mora *et al.*, 2020 ▸). We ensured that the crystals were kept centered during data collection, and due to the inherent nature of crystal-to-crystal variation in serial crystallography we report the estimated DWD as an average value. All data-collection statistics are reported in Table 1 ▸.

### 2.4. Crystallographic data processing

Diffraction data were processed using both *XDS* (Kabsch, 2010 ▸) and *DIALS* (Winter *et al.*, 2022 ▸).

For the *XDS* pipeline, inputs were created using a custom script that ran the generate_XDS.INP script for each wedge in each chip. As noted above, each wedge consists of ten frames, with the first six used for processing and the last four blank frames excluded by the script. The script generated XDS.INP files and ran the parallelized version of *XDS* (xds_par) for each wedge. See Supplementary Fig. S2 for the list of steps run by xds_par. Space group *P*3_1_21 was explicitly enforced. Subsequent scaling and merging of integrated intensities across all wedges across all used chips was performed using *XSCALE*. *XDSCONV* was used to convert the unmerged XSCALE.HKL file to a merged .hkl file and then to a merged .mtz file, which was used for subsequent processes.

For the *DIALS* pipeline, data were imported using *dials.import* followed by spot-finding using *dials.find_spots*. A custom Python script was used to identify wedges as sets of consecutive frames with at least 20 identified diffraction spots, excluding blank images (see above), with the result stored in a single .expt and .refl file. Data for all wedges were indexed using *dials.index* with the flags joint_index=false and beam.fix=all detector.fix=all. The results were refined using *dials.refine*, and *dials.split_experiments* was run using refined.* files to create split_*.refl and split_*.expt files for individual wedges. Within a separate directory for each wedge, *dials.integrate* was run. Successfully integrated wedges were scaled and merged using *xia*2.*multiplex* (Gildea *et al.*, 2022 ▸), using flags to impose the space group (symmetry.space_group=P3121), completeness (min_completeness=0.95) and resolution (d_min=2.40). The resulting merged .mtz file was used for subsequent processes.

For Table 1 ▸ and Table 2 ▸, Wilson *B* values were obtained by running *phenix.table_one* with the merged data, and some statistics not initially provided by *XDS* were obtained by running *phenix.merging_statistics* with the final unmerged data from* XSCALE*.

### 2.5. Structure refinement and modeling

The structure was solved using molecular replacement via *Phaser* (McCoy *et al.*, 2007 ▸) using PDB entry 1t49 (with waters and the allosteric inhibitor excluded; Wiesmann *et al.*, 2004 ▸) as the search model. Iterative rounds of refinement were performed using *phenix.refine* (Adams *et al.*, 2010 ▸) and *Coot* (Emsley *et al.*, 2010 ▸) and the model quality was validated using *MolProbity* (Chen *et al.*, 2010 ▸; Williams *et al.*, 2018 ▸). Data-reduction and refinement statistics are reported in Table 2 ▸. Figures were prepared using *PyMOL* version 2.5 (Schrödinger) via .pml scripting.

### 2.6. Data availability

Model coordinates and structure factors have been deposited in the Protein Data Bank as PDB entry 8du7. Raw diffraction images are available from SBGrid at https://doi.org/10.15785/SBGRID/961.

## 3. Results

To determine a room-temperature serial synchrotron crystallographic structure of PTP1B, we used a recently introduced fixed-target serial sample-support system, including multifaceted chips that are loaded with microcrystals in a custom humidified environment (Illava *et al.*, 2021 ▸; Supplementary Fig. S1). Using this system on the ID7B2 beamline at MacCHESS, we loaded six chips with PTP1B crystals in the previously characterized *P*3_1_21 space group (Pedersen *et al.*, 2004 ▸; Keedy *et al.*, 2018 ▸). Across these chips, we collected a total of 1297 partial data sets (wedges) constituting 1.2–3.0° each. The diffraction-weighted dose for each crystal in this experiment was estimated using *RADDOSE*-3*D* (Bury *et al.*, 2018 ▸) to be <35.1 kGy per crystal (see Section 2.3), suggesting an absence of substantial radiation damage.

To ensure that our data were processed robustly, we used two pipelines in parallel, *XDS* (Kabsch, 2010 ▸) and *DIALS* (Winter *et al.*, 2022 ▸), and compared the results. The two pipelines involved different software, but had a similar overall logic: splitting into wedges, spot-finding, indexing and integration for each wedge, and scaling and merging across all wedges (Supplementary Fig. S2). Both pipelines yielded similar numbers of successfully processed wedges across the six chips, with no discernible pattern as to which wedges or chips were best processed by either method (Supplementary Table S1). The overall ‘hit rate’ for *XDS* was ∼10%, with 129 successfully processed wedges for *XDS* (and similar for *DIALS*) out of a total of 1297 collected wedges. This efficiency could likely be improved with experimental optimization, which was not performed here.

**Figure 1:**
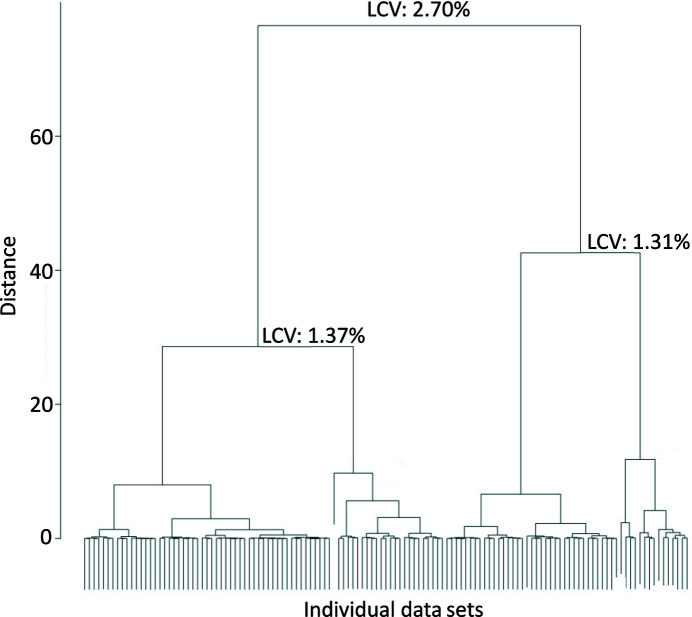
Hierarchical clustering analysis of PTP1B SSX data. *BLEND* analysis of all individual data sets (wedges, horizontal axis) based on linear cell variation (LCV; Foadi *et al.*, 2013 ▸) results in a dendrogram illustrating a series of hierarchical clusters that are separated by the Ward distance metric (vertical axis).

Analysis of the correlation coefficients between all of the unmerged wedges with *XSCALE_ISOCLUSTER* (Diederichs, 2017 ▸) indicated the existence of only one cluster, thus obviating the need for merging separate isomorphous clusters of subsets of the data. We also performed a hierarchical clustering analysis using *BLEND* based on linear cell variation (LCV), a metric based on unit-cell parameters (Foadi *et al.*, 2013 ▸; Fig. 1 ▸). Hierarchical clusters are identified with LCVs of 2.70% for the overall main cluster and 1.31% and 1.37% for the first two subclusters. Upon merging within these two subclusters, one yielded low completeness (67%) and thus was not pursued further. The other subcluster yielded higher completeness (97%) and a structural model that was nearly identical to that obtained from all data (C^α^ r.m.s.d. of 0.10 Å), with no noticeable differences in the key structural sites featured throughout this manuscript. These observations suggest a nonzero but relatively low level of non-isomorphism with limited impact on the structural state of PTP1B.

We therefore used all successfully processed wedges for *XDS* and for *DIALS*. These data sets both have similarly good overall statistics after scaling and merging (Table 1 ▸). We then performed molecular replacement and iterative model building and refinement for both data sets (Table 2 ▸). The *XDS*-derived and *DIALS*-derived models are very similar (C^α^ r.m.s.d. of 0.21 Å; Supplementary Fig. S3). We therefore focused on the *XDS* model for the remainder of this work.

**Figure 2:**
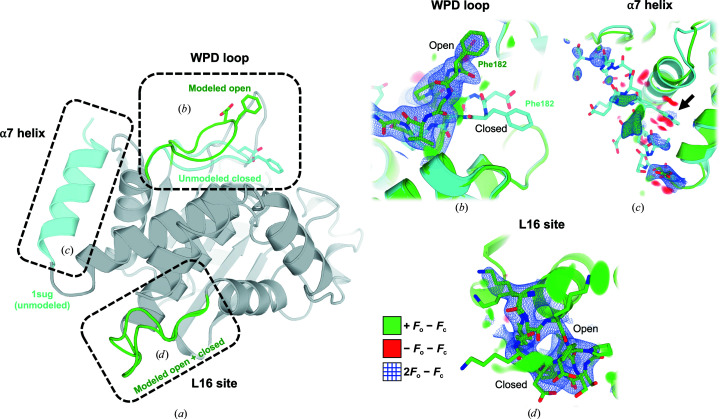
Conformational heterogeneity across the PTP1B allosteric network in an SSX RT structure. (*a*) Overview of the key sites in PTP1B featured in (*b*)–(*d*). (*b*) The active-site WPD loop is best modeled as adopting only the open conformation, based on 2*F*_o_ − *F*_c_ electron density contoured at 1σ (blue) and positive/negative *F*_o_ − *F*_c_ difference electron density contoured at ±3.0σ (green/red). The closed conformation of the WPD loop as modeled in PDB entry 1sug (transparent cyan) is shown for comparison. See also Supplementary Fig. S4 (dual conformation). (*c*) The α7 helix is best modeled as disordered, based on 2*F*_o_ − *F*_c_ density at 1σ and *F*_o_ − *F*_c_ density at ±3.0σ. The ordered conformation of α7 from PDB entry 1sug is shown for comparison. Note the absence of strong density for the Trp291 ‘anchor’ that can occupy the allosteric BB site (arrow). See also Supplementary Fig. S5 (α7 refined). (*d*) In contrast to the WPD loop and α7, Loop 16 definitively adopts alternate conformations with similar occupancies, based on 2*F*_o_ − *F*_c_ density at 1σ and *F*_o_ − *F*_c_ density at ±3.0σ. See also Supplementary Fig. S5 (omit maps).

Using this final model, we next inspected the conformational ensemble of the protein at several key sites (Fig. 2 ▸).

Firstly, the active-site WPD loop adopts the open conformation (Fig. 2 ▸ *b*), which is known to dominate in solution (Whittier *et al.*, 2013 ▸). There is some residual positive *F*_o_ − *F*_c_ electron density below this open conformation near the location of the closed conformation seen in previous structures (Pedersen *et al.*, 2004 ▸). However, refinement with a dual-conformation model as used previously (Keedy *et al.*, 2018 ▸) resulted in an absence of 2*F*_o_ − *F*_c_ density above 0.7σ for the closed conformation (Supplementary Fig. S4), arguing against this interpretation of the data. Some residual density is detectable below the WPD loop and may be attributable to the complex water network of the active site (Pedersen *et al.*, 2004 ▸), although the moderate resolution of our data set limits our ability to interpret the details of this network.

A second key site is the α7 helix, which has been established by a variety of methods as a key allosteric hub in PTP1B (Olmez & Alakent, 2011 ▸; Krishnan *et al.*, 2014 ▸; Choy *et al.*, 2017 ▸; Keedy *et al.*, 2018 ▸) as well as in its closest homolog TCPTP (Singh *et al.*, 2021 ▸). In our SSX structure of PTP1B, the α7 helix cannot be confidently modeled in the ordered conformation, and thus is best left disordered as in many previous open-state structures (Fig. 2 ▸ *c*). An attempt to model the ordered conformation results in detectable but weak electron-density support (Supplementary Fig. S5) and inflated *B* factors of >100 Å^2^ (compared with ∼40–60 Å^2^ for the more fully ordered α7 in PDB entry 1sug).

A third key site in the protein, Loop 16, is the eponymous loop of the reported allosteric L16 site (Keedy *et al.*, 2018 ▸), which also involves the adjacent protein N-terminus and the C-terminus of the α6 helix as it transitions to α7. The L16 site constitutes a cryptic site (Cimermancic *et al.*, 2016 ▸) that only accommodates ligands in its open conformation, as discovered in a high-throughput crystallographic small-molecule fragment screen (Keedy *et al.*, 2018 ▸), adding to its potential value as a targetable allosteric site in PTP1B. In our SSX structure, unlike the WPD loop and the α7 helix, PTP1B clearly adopts alternate conformations for Loop 16 in the open and closed states (Fig. 2 ▸ *d*). These conformations have a similar occupancy in the refined model (46% open, 54% closed), suggesting that they are approximately isoenergetic in our experimental conditions. Omit maps of either conformation result in convincing difference density features, confirming the simultaneous presence of both states in the crystal (Supplementary Fig. S6).

**Figure 3:**
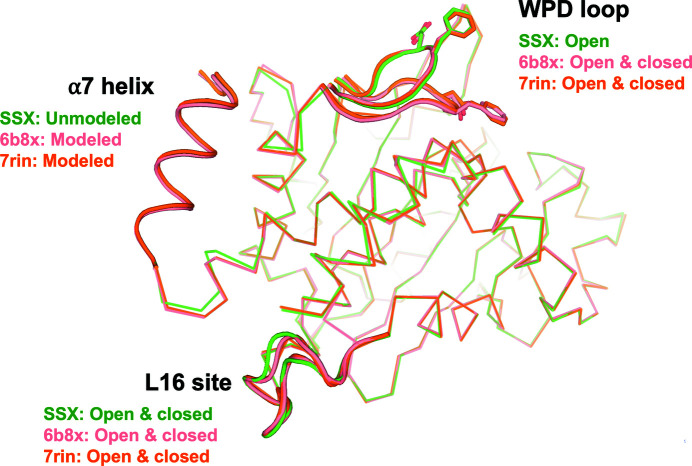
The SSX RT structure and previous near-RT structures of PTP1B have different patterns of conformational heterogeneity across the allosteric network. Overlaid are our SSX structure (green), PDB entry 6b8x (pink) and PDB entry 7rin (orange).

These observations from our SSX structure are in contrast to the previous report of an allosteric network in PTP1B with coupled opening of the WPD loop, opening of the L16 site and disordering of the α7 helix (Keedy *et al.*, 2018 ▸). This concept is embodied in PDB entry 6b8x (and PDB entry 6b8t), which have open/closed alternate conformations for the WPD loop, open/closed alternate conformations for the L16 site and a quasi-ordered partial-occupancy α7 (Fig. 3 ▸). Another recently deposited RT structure of apo PTP1B, PDB entry 7rin (Greisman *et al.*, 2022 ▸), agrees with PDB entry 6b8x in these respects, adopting the same open/closed WPD and L16 site coupling and partial occupancy of α7 (Fig. 3 ▸). Both PDB entries 6b8x and 7rin were expressed using the same 1–321 C32S/C92V double-mutation construct (referred to as WT*) as used in our study, and were crystallized under similar conditions, including pH. Further, they were found to belong to the same space group as our SSX structure, with extremely similar unit-cell dimensions. PDB entries 7rin and 6b8x do differ in the fact that the crystals used to obtain PDB entry 6b8x include a high concentration of glycerol, subsequently leading to glycerol molecules bound to the active site. Despite the fact that glycerol bound to the active site of PTP1B may bias the conformational ensemble of the protein, PDB entry 6b8x still adopts a nearly identical conformation to the apo structure PDB entry 7rin. The more discernible conformational heterogeneity of these previous structures compared with our structure may be related to their higher resolution (1.74 Å for PDB entry 6b8x and 1.85 Å for PDB entry 7rin) compared with our SSX structure (2.40 Å). Nevertheless, the differences between our SSX structure and previous RT WT (or similar) structures of PTP1B suggest that the active-site WPD loop and the allosteric L16 site may not be as strictly coupled as previously imagined.

The remaining apparently near-RT data set, PDB entry 2cm2, is at even higher resolution (1.50 Å), yet features a single-conformation open WPD loop, an open L16 site and a disordered α7 (Ala *et al.*, 2006 ▸), which is distinct from our previous RT WT (or WT*) structures, including our SSX structure reported here. In theory, these differences could be due to the different space group of PDB entry 2cm2 (*P*2_1_2_1_2_1_) relative to the other aforementioned structures (*P*3_1_21). However, upon a closer inspection of the PDB metadata compared with the crystallographic statistics presented in the manuscript, the data for PDB entry 2cm2 were actually collected at ∼93 K and not at 277 K as reported in the PDB. Unfortunately, the *P*2_1_2_1_2_1_ PTP1B data set from the same study that was collected at 277 K was not deposited in the PDB. It therefore remains unknown whether the different space group, unit cell and crystal contacts seen in PDB entry 2cm2 may explain its collapsed conformational ensemble at these sites, and whether these features would carry over at elevated temperatures. Unfortunately, structure factors are not available for PDB entry 2cm2, so we cannot interrogate its electron-density map for signs of ‘hidden’ unmodeled alternate conformations (Lang *et al.*, 2010 ▸), as detected previously for the cryogenic data set PDB entry 1sug (Keedy *et al.*, 2018 ▸).

## 4. Discussion

Here, we present the first SSX structure of a human phosphatase, in this case PTP1B. The serial sample-support system used here (Illava *et al.*, 2021 ▸) is flexible, allowing data collection at RT or cryo using standard X-ray beamline goniometers with minimized radiation damage. It thus represents a positive addition to the expanding toolkit for RT crystallography experiments (Fischer, 2021 ▸).

Unsurprisingly, our SSX structure of PTP1B is broadly similar to previous single-crystal structures in this and other space groups. Nevertheless, demonstrating the feasibility of serial experiments for this biomedically central protein is a valuable step. Moreover, our results add nuance to the previously reported picture of the allosteric network in PTP1B, in which opening of the WPD loop, disordering of the α7 helix and opening of Loop 16 are highly correlated (Keedy *et al.*, 2018 ▸). In the SSX structure, it appears that the expected coupling between the WPD loop and α7 is present: the former is open (Fig. 2 ▸ *b*, Supplementary Fig. S4) and the latter is disordered (Fig. 2 ▸ *c*, Supplementary Fig. S5). However, Loop 16 adopts both open and closed conformations (Fig. 2 ▸ *d*), suggesting that coupling between the L16 site and the other two areas may not be as tight as previously hypothesized. This reframing is reminiscent of how the active-site side-chain network of the proline isomerase CypA was previously viewed as thoroughly coupled (Fraser *et al.*, 2009 ▸), but was reinterpreted as involving hierarchical coupling on the basis of a multitemperature crystallography series (Keedy *et al.*, 2015 ▸). Such observations highlight the need for multi-data-set and serial approaches to crystallography and accompanying multiconformer modeling, as these approaches can be used to average out single-crystal bias. This allows effective elucidation and modeling of conformationally diverse areas that may only be represented as a single conformation in single-crystal RT crystallography, bettering our ability to infer allosteric mechanisms in proteins (Keedy, 2019 ▸).

Although we observe different coupling between sites in our SSX structure versus previous RT apo structures, some caveats should be considered that complicate this interpretation. Firstly, the resolution of our structure is only moderate (2.40 Å) when compared with previous single-crystal structures (<2 Å), although this could likely be improved by optimizing our crystals for serial diffraction, which we did not undertake. Secondly, although the unit cell and the crystal contacts in our structure are the same as in PDB entries 6b8x and 7rin, they are different from those in PDB entry 2cm2, the data for which were in reality collected at cryogenic temperature (Ala *et al.*, 2006 ▸) and not at near-RT as reported in the PDB metadata. The accompanying near-RT structure to PDB entry 2cm2 was not deposited in the PDB, but its existence highlights the potential of exploring variable-temperature crystallography in different crystal forms. Moving forward, such crystal lattice differences can present opportunities to learn from differential localized perturbations to surface regions of proteins; this is conceptually similar to how crystallographic alternate conformations were often viewed as a nuisance during model building and refinement, but can now be exploited to learn about biological function (Fraser *et al.*, 2009 ▸).

In closing, we look forward to the ongoing development of serial crystallography approaches to enable powerful interrogations of functional motions in PTP1B and other systems, including time-resolved X-ray crystallography with on-chip mixing (Mehrabi *et al.*, 2020 ▸) or photoactivation via caged compounds (Monteiro *et al.*, 2021 ▸) at synchrotrons or XFELs. Such experiments may enjoy synergy with complementary approaches to interrogate protein motions such as crystal diffuse scattering (Wall *et al.*, 2018 ▸; Meisburger *et al.*, 2020 ▸) and solution hydrogen–deuterium exchange mass spectrometry (Kaltashov *et al.*, 2009 ▸).

## Supplementary Material

X-ray diffraction data from protein tyrosine phosphatase 1B, source of 8du7 structure: https://doi.org/10.15785/SBGRID/961

PDB reference: room-temperature serial synchrotron crystallographic structure of apo PTP1B, 8du7

Supplementary Table and Figures. DOI: 10.1107/S2053230X22011645/rs5005sup1.pdf

## Figures and Tables

**Table 1:** Crystallographic statistics for *XDS* and *DIALS* data reduction Values in parentheses are for the highest resolution bin.

Method	*XDS*	*DIALS*
Resolution (Å)	44.67–2.40 (2.53–2.40)	105.5–2.40 (2.53–2.40)
Completeness (%)	99.7 (99.7)	99.82 (100.0)
Multiplicity	11.45 (11.06)	12.10 (12.68)
〈*I*/σ(*I*)〉	4.62 (1.08)	4.5 (0.8)
*R*_merge_(*I*)	0.661 (4.23)	0.938 (5.921)
*R*_meas_(*I*)	0.693 (4.754)	0.980 (6.166)
*R*_p.i.m._(*I*)	0.201 (1.307)	0.272 (1.666)
CC_1/2_	0.881 (0.135)	0.929 (0.218)
Wilson *B* factor (Å^2^)	47.57	40.81
Total observations	223455 (15663)	234336 (11935)
Unique observations	19515 (1416)	19359 (941)
Space group	*P*3_1_21	*P*3_1_21
*a*, *b*, *c* (Å)	89.35, 89.35, 105.76	89.06, 89.06, 105.48
α, β, γ (°)	90, 90, 120	90, 90, 120

**Table 2:** Crystallographic statistics for *XDS* data reduction and structural modeling Values in parentheses are for the highest resolution bin.

PDB code	8du7
Method	*XDS*
Resolution (Å)	44.67–2.40
Completeness (%)	99.7 (99.7)
Multiplicity	11.45 (11.06)
〈*I*/σ(*I*)〉	4.62 (1.08)
*R*_merge_(*I*)	0.661 (4.23)
*R*_meas_(*I*)	0.693 (4.754)
*R*_p.i.m._(*I*)	0.201 (1.307)
CC_1/2_	0.881 (0.135)
Wilson *B* factor (Å^2^)	47.57
Total observations	223455 (15663)
Unique observations	19515 (1416)
Space group	*P*3_1_2
*a*, *b*, *c* (Å)	89.35, 89.35, 105.76
α, β, γ (°)	90, 90, 120
Solvent content (%)	62.64
*R*_work_	0.195
*R*_free_	0.237
R.m.s.d., bond lengths (Å)	0.013
R.m.s.d., angles (°)	1.33
Ramachandran outliers (%)	0.35
Ramachandran favored (%)	92.55
Clashscore	6.06
*MolProbity* score	2.12

## References

[bb1] Adams, P. D., Afonine, P. V., Bunkóczi, G., Chen, V. B., Davis, I. W., Echols, N., Headd, J. J., Hung, L.-W., Kapral, G. J., Grosse-Kunstleve, R. W., McCoy, A. J., Moriarty, N. W., Oeffner, R., Read, R. J., Richardson, D. C., Richardson, J. S., Terwilliger, T. C. & Zwart, P. H. (2010). *Acta Cryst.* D**66**, 213–221. 10.1107/S0907444909052925 PMC2815670 20124702

[bb2] Ala, P. J., Gonneville, L., Hillman, M. C., Becker-Pasha, M., Wei, M., Reid, B. G., Klabe, R., Yue, E. W., Wayland, B., Douty, B., Polam, P., Wasserman, Z., Bower, M., Combs, A. P., Burn, T. C., Hollis, G. F. & Wynn, R. (2006). *J. Biol. Chem.* **281**, 32784–32795. 10.1074/jbc.M606873200 16916797

[bb3] Bedem, H. van den, Bhabha, G., Yang, K., Wright, P. E. & Fraser, J. S. (2013). *Nat. Methods*, **10**, 896–902. 10.1038/nmeth.2592 PMC3760795 23913260

[bb4] Berman, H. M., Westbrook, J., Feng, Z., Gilliland, G., Bhat, T. N., Weissig, H., Shindyalov, I. N. & Bourne, P. E. (2000). *Nucleic Acids Res.* **28**, 235–242. 10.1093/nar/28.1.235 PMC102472 10592235

[bb5] Bury, C. S., Brooks-Bartlett, J. C., Walsh, S. P. & Garman, E. F. (2018). *Protein Sci.* **27**, 217–228. 10.1002/pro.3302 PMC5734275 28921782

[bb6] Chen, V. B., Arendall, W. B., Headd, J. J., Keedy, D. A., Immormino, R. M., Kapral, G. J., Murray, L. W., Richardson, J. S. & Richardson, D. C. (2010). *Acta Cryst.* D**66**, 12–21. 10.1107/S0907444909042073 PMC2803126 20057044

[bb7] Choy, M. S., Li, Y., Machado, L. E. S. F., Kunze, M. B. A., Connors, C. R., Wei, X., Lindorff-Larsen, K., Page, R. & Peti, W. (2017). *Mol. Cell*, **65**, 644–658. 10.1016/j.molcel.2017.01.014 PMC5325675 28212750

[bb8] Cimermancic, P., Weinkam, P., Rettenmaier, T. J., Bichmann, L., Keedy, D. A., Woldeyes, R. A., Schneidman-Duhovny, D., Demerdash, O. N., Mitchell, J. C., Wells, J. A., Fraser, J. S. & Sali, A. (2016). *J. Mol. Biol.* **428**, 709–719. 10.1016/j.jmb.2016.01.029 PMC4794384 26854760

[bb9] Diederichs, K. (2017). *Acta Cryst.* D**73**, 286–293. 10.1107/S2059798317000699 PMC5379934 28375141

[bb10] Diederichs, K. & Wang, M. (2017). *Methods Mol. Biol.* **1607**, 239–272. 10.1007/978-1-4939-7000-1_10 28573576

[bb88] Drake, P. G. & Posner, B. I. (1998). *Mol. Cell. Biochem.* **182**, 79–89. 9609117

[bb11] Ebrahim, A., Appleby, M. V., Axford, D., Beale, J., Moreno-Chicano, T., Sherrell, D. A., Strange, R. W., Hough, M. A. & Owen, R. L. (2019). *Acta Cryst.* D**75**, 151–159. 10.1107/S2059798318010240 PMC6400251 30821704

[bb12] Ebrahim, A., Moreno-Chicano, T., Appleby, M. V., Chaplin, A. K., Beale, J. H., Sherrell, D. A., Duyvesteyn, H. M. E., Owada, S., Tono, K., Sugimoto, H., Strange, R. W., Worrall, J. A. R., Axford, D., Owen, R. L. & Hough, M. A. (2019). *IUCrJ*, **6**, 543–551. 10.1107/S2052252519003956 PMC6608622 31316799

[bb13] Emsley, P., Lohkamp, B., Scott, W. G. & Cowtan, K. (2010). *Acta Cryst.* D**66**, 486–501. 10.1107/S0907444910007493 PMC2852313 20383002

[bb14] Fischer, M. (2021). *Q. Rev. Biophys.* **54**, e1. 10.1017/S0033583520000128 33413726

[bb15] Fischer, M., Shoichet, B. K. & Fraser, J. S. (2015). *ChemBioChem*, **16**, 1560–1564. 10.1002/cbic.201500196 PMC4539595 26032594

[bb16] Foadi, J., Aller, P., Alguel, Y., Cameron, A., Axford, D., Owen, R. L., Armour, W., Waterman, D. G., Iwata, S. & Evans, G. (2013). *Acta Cryst.* D**69**, 1617–1632. 10.1107/S0907444913012274 PMC3727331 23897484

[bb17] Fraser, J. S., Clarkson, M. W., Degnan, S. C., Erion, R., Kern, D. & Alber, T. (2009). *Nature*, **462**, 669–673. 10.1038/nature08615 PMC2805857 19956261

[bb18] Fraser, J. S., van den Bedem, H., Samelson, A. J., Lang, P. T., Holton, J. M., Echols, N. & Alber, T. (2011). *Proc. Natl Acad. Sci. USA*, **108**, 16247–16252. 10.1073/pnas.1111325108 PMC3182744 21918110

[bb19] Friedman, A. J., Liechty, E. T., Kramer, L., Sarkar, A., Fox, J. M. & Shirts, M. R. (2022). *J. Phys. Chem. B*, **126**, 8427–8438. 10.1021/acs.jpcb.2c05423 PMC10040085 36223525

[bb20] Garman, E. F. & Nave, C. (2009). *J. Synchrotron Rad.* **16**, 129–132.

[bb21] Gildea, R. J., Beilsten-Edmands, J., Axford, D., Horrell, S., Aller, P., Sandy, J., Sanchez-Weatherby, J., Owen, C. D., Lukacik, P., Strain-Damerell, C., Owen, R. L., Walsh, M. A. & Winter, G. (2022). *Acta Cryst.* D**78**, 752–769. 10.1107/S2059798322004399 PMC9159281 35647922

[bb22] Greisman, J. B., Dalton, K. M., Sheehan, C. J., Klureza, M. A., Kurinov, I. & Hekstra, D. R. (2022). *Acta Cryst.* D**78**, 986–996. 10.1107/S2059798322006799 PMC9344477 35916223

[bb23] Hansen, S. K., Cancilla, M. T., Shiau, T. P., Kung, J., Chen, T. & Erlanson, D. A. (2005). *Biochemistry*, **44**, 7704–7712. 10.1021/bi047417s 15909985

[bb24] Hirata, K., Shinzawa-Itoh, K., Yano, N., Takemura, S., Kato, K., Hatanaka, M., Muramoto, K., Kawahara, T., Tsukihara, T., Yamashita, E., Tono, K., Ueno, G., Hikima, T., Murakami, H., Inubushi, Y., Yabashi, M., Ishikawa, T., Yamamoto, M., Ogura, T., Sugimoto, H., Shen, J.-R., Yoshikawa, S. & Ago, H. (2014). *Nat. Methods*, **11**, 734–736. 10.1038/nmeth.2962 24813624

[bb25] Illava, G., Jayne, R., Finke, A. D., Closs, D., Zeng, W., Milano, S. K., Huang, Q., Kriksunov, I., Sidorenko, P., Wise, F. W., Zipfel, W. R., Apker, B. A. & Thorne, R. E. (2021). *Acta Cryst.* D**77**, 628–644. 10.1107/S2059798321001868 PMC8098472 33950019

[bb26] Kabsch, W. (2010). *Acta Cryst.* D**66**, 125–132. 10.1107/S0907444909047337 PMC2815665 20124692

[bb27] Kaltashov, I. A., Bobst, C. E. & Abzalimov, R. R. (2009). *Anal. Chem.* **81**, 7892–7899. 10.1021/ac901366n PMC2805115 19694441

[bb28] Keedy, D. A. (2019). *Acta Cryst.* D**75**, 123–137. 10.1107/S2059798318017941 PMC6400254 30821702

[bb29] Keedy, D. A., Hill, Z. B., Biel, J. T., Kang, E., Rettenmaier, T. J., Brandão-Neto, J., Pearce, N. M., von Delft, F., Wells, J. A. & Fraser, J. S. (2018). *eLife*, **7**, e36307. 10.7554/eLife.36307 PMC6039181 29877794

[bb30] Keedy, D. A., Kenner, L. R., Warkentin, M., Woldeyes, R. A., Hopkins, J. B., Thompson, M. C., Brewster, A. S., Van Benschoten, A. H., Baxter, E. L., Uervirojnangkoorn, M., McPhillips, S. E., Song, J., Alonso-Mori, R., Holton, J. M., Weis, W. I., Brunger, A. T., Soltis, S. M., Lemke, H., Gonzalez, A., Sauter, N. K., Cohen, A. E., van den Bedem, H., Thorne, R. E. & Fraser, J. S. (2015). *eLife*, **4**, e07574. 10.7554/eLife.07574 PMC4721965 26422513

[bb31] Keedy, D. A., van den Bedem, H., Sivak, D. A., Petsko, G. A., Ringe, D., Wilson, M. A. & Fraser, J. S. (2014). *Structure*, **22**, 899–910. 10.1016/j.str.2014.04.016 PMC4082491 24882744

[bb32] Krishnan, N., Konidaris, K. F., Gasser, G. & Tonks, N. K. (2018). *J. Biol. Chem.* **293**, 1517–1525. 10.1074/jbc.C117.819110 PMC5798283 29217773

[bb33] Krishnan, N., Koveal, D., Miller, D. H., Xue, B., Akshinthala, S. D., Kragelj, J., Jensen, M. R., Gauss, C.-M., Page, R., Blackledge, M., Muthuswamy, S. K., Peti, W. & Tonks, N. K. (2014). *Nat. Chem. Biol.* **10**, 558–566. 10.1038/nchembio.1528 PMC4062594 24845231

[bb34] Krishnan, N., Krishnan, K., Connors, C. R., Choy, M. S., Page, R., Peti, W., Van Aelst, L., Shea, S. D. & Tonks, N. K. (2015). *J. Clin. Invest.* **125**, 3163–3177. 10.1172/JCI80323 PMC4563751 26214522

[bb35] Lang, P. T., Ng, H.-L., Fraser, J. S., Corn, J. E., Echols, N., Sales, M., Holton, J. M. & Alber, T. (2010). *Protein Sci.* **19**, 1420–1431. 10.1002/pro.423 PMC2974833 20499387

[bb36] McCoy, A. J., Grosse-Kunstleve, R. W., Adams, P. D., Winn, M. D., Storoni, L. C. & Read, R. J. (2007). *J. Appl. Cryst.* **40**, 658–674. 10.1107/S0021889807021206 PMC2483472 19461840

[bb37] Mehrabi, P., Müller-Werkmeister, H. M., Leimkohl, J.-P., Schikora, H., Ninkovic, J., Krivokuca, S., Andriček, L., Epp, S. W., Sherrell, D., Owen, R. L., Pearson, A. R., Tellkamp, F., Schulz, E. C. & Miller, R. J. D. (2020). *J. Synchrotron Rad.* **27**, 360–370. 10.1107/S1600577520000685 PMC7064102 32153274

[bb38] Meisburger, S. P., Case, D. A. & Ando, N. (2020). *Nat. Commun.* **11**, 1271. 10.1038/s41467-020-14933-6 PMC7062842 32152274

[bb39] Milano, S. K., Huang, Q., Nguyen, T.-T. T., Ramachandran, S., Finke, A., Kriksunov, I., Schuller, D. J., Szebenyi, D. M., Arenholz, E., McDermott, L. A., Sukumar, N., Cerione, R. A. & Katt, W. P. (2022). *J. Biol. Chem.* **298**, 101535. 10.1016/j.jbc.2021.101535 PMC8784640 34954143

[bb40] Montalibet, J. & Kennedy, B. P. (2005). *Drug. Discov. Today Ther. Strateg.* **2**, 129–135.

[bb41] Monteiro, D. C. F., Amoah, E., Rogers, C. & Pearson, A. R. (2021). *Acta Cryst.* D**77**, 1218–1232. 10.1107/S2059798321008809 PMC8489231 34605426

[bb42] Mora, E. de la, Coquelle, N., Bury, C. S., Rosenthal, M., Holton, J. M., Carmichael, I., Garman, E. F., Burghammer, M., Colletier, J.-P. & Weik, M. (2020). *Proc. Natl Acad. Sci.* **117**, 4142–4151. 10.1073/pnas.1821522117 PMC7049125 32047034

[bb43] Moreno-Chicano, T., Ebrahim, A., Axford, D., Appleby, M. V., Beale, J. H., Chaplin, A. K., Duyvesteyn, H. M. E., Ghiladi, R. A., Owada, S., Sherrell, D. A., Strange, R. W., Sugimoto, H., Tono, K., Worrall, J. A. R., Owen, R. L. & Hough, M. A. (2019). *IUCrJ*, **6**, 1074–1085. 10.1107/S2052252519011655 PMC6830213 31709063

[bb44] Olmez, E. O. & Alakent, B. (2011). *J. Biomol. Struct. Dyn.* **28**, 675–693. 10.1080/07391102.2011.10508599 21294582

[bb45] Owen, R. L., Axford, D., Sherrell, D. A., Kuo, A., Ernst, O. P., Schulz, E. C., Miller, R. J. D. & Mueller-Werkmeister, H. M. (2017). *Acta Cryst.* D**73**, 373–378. 10.1107/S2059798317002996 PMC5379936 28375148

[bb46] Pearce, N. M., Krojer, T., Bradley, A. R., Collins, P., Nowak, R. P., Talon, R., Marsden, B. D., Kelm, S., Shi, J., Deane, C. M. & von Delft, F. (2017). *Nat. Commun.* **8**, 15123. 10.1038/ncomms15123 PMC5413968 28436492

[bb47] Pedersen, A. K., Peters, G. H., Møller, K. B., Iversen, L. F. & Kastrup, J. S. (2004). *Acta Cryst.* D**60**, 1527–1534. 10.1107/S0907444904015094 15333922

[bb49] Singh, J. P., Lin, M.-J., Hsu, S.-F., Peti, W., Lee, C.-C. & Meng, T.-C. (2021). *Biochemistry*, **60**, 3856–3867. 10.1021/acs.biochem.1c00519 34910875

[bb50] Stanford, S. M. & Bottini, N. (2017). *Trends Pharmacol. Sci.* **38**, 524–540. 10.1016/j.tips.2017.03.004 PMC5494996 28412041

[bb51] Tonks, N. K. & Muthuswamy, S. K. (2007). *Cancer Cell*, **11**, 214–216. 10.1016/j.ccr.2007.02.022 17349579

[bb52] Vieira, M. N. N., Lyra, E., Silva, N. M., Ferreira, S. T. & De Felice, F. G. (2017). *Front. Aging Neurosci.* **9**, 7. 10.3389/fnagi.2017.00007 PMC5281585 28197094

[bb53] Wall, M. E., Wolff, A. M. & Fraser, J. S. (2018). *Curr. Opin. Struct. Biol.* **50**, 109–116. 10.1016/j.sbi.2018.01.009 PMC6078797 29455056

[bb54] Warkentin, M., Hopkins, J. B., Badeau, R., Mulichak, A. M., Keefe, L. J. & Thorne, R. E. (2013). *J. Synchrotron Rad.* **20**, 7–13. 10.1107/S0909049512048303 PMC3526918 23254651

[bb55] Whittier, S. K., Hengge, A. C. & Loria, J. P. (2013). *Science*, **341**, 899–903. 10.1126/science.1241735 PMC4078984 23970698

[bb56] Wiesmann, C., Barr, K. J., Kung, J., Zhu, J., Erlanson, D. A., Shen, W., Fahr, B. J., Zhong, M., Taylor, L., Randal, M., McDowell, R. S. & Hansen, S. K. (2004). *Nat. Struct. Mol. Biol.* **11**, 730–737. 10.1038/nsmb803 15258570

[bb57] Williams, C. J., Headd, J. J., Moriarty, N. W., Prisant, M. G., Videau, L. L., Deis, L. N., Verma, V., Keedy, D. A., Hintze, B. J., Chen, V. B., Jain, S., Lewis, S. M., Arendall, W. B., Snoeyink, J., Adams, P. D., Lovell, S. C., Richardson, J. S. & Richardson, J. S. (2018). *Protein Sci.* **27**, 293–315. 10.1002/pro.3330 PMC5734394 29067766

[bb58] Winter, G., Beilsten-Edmands, J., Devenish, N., Gerstel, M., Gildea, R. J., McDonagh, D., Pascal, E., Waterman, D. G., Williams, B. H. & Evans, G. (2022). *Protein Sci.* **31**, 232–250. 10.1002/pro.4224 PMC8740827 34747533

[bb59] Xu, Q., Wu, N., Li, X., Guo, C., Li, C., Jiang, B., Wang, H. & Shi, D. (2019). *Cell Death Dis.* **10**, 874. 10.1038/s41419-019-2073-4 PMC6864061 31745071

[bb60] Zhang, S. & Zhang, Z.-Y. (2007). *Drug Discov. Today*, **12**, 373–381. 10.1016/j.drudis.2007.03.011 17467573

[bb61] Zhang, Z.-Y. (2001). *Curr. Opin. Chem. Biol.* **5**, 416–423.

[bb62] Zhang, Z.-Y. & Lee, S.-Y. (2003). *Expert Opin. Investig. Drugs*, **12**, 223–233. 10.1517/13543784.12.2.223 12556216

