# Supplementary material for: Room-temperature serial synchrotron crystallography of the human phosphatase PTP1B
Source: Acta Crystallogr F Struct Biol Commun. 2023 Jan 1;79(Pt 1):23–30. doi: 10.1107/S2053230X22011645 (PMC9813971; doi:10.1107/S2053230X22011645)
Supplement: Supplementary file 1 [file f-79-00023-sup1.pdf]

Acta Cryst F STRUCTURAL BIOLOGY COMMUNICATIONS

Volume 79 (2023)

Supporting information for article:

Room-temperature serial synchrotron crystallography of the human phosphatase PTP1B

Shivani Sharma, Ali Ebrahim and Daniel A. Keedy

Table S1 Successfully integrated wedges per chip.

The last row reports the number of wedges that were successfully integrated by both XDS and DIALS.

	Chip 1	Chip 2	Chip 3	Chip 4	Chip 5	Chip 6	Total
XDS	22	18	18	14	20	37	129
DIALS	16	20	17	7	23	36	121
XDS + DIALS	14	15	15	6	16	24	90

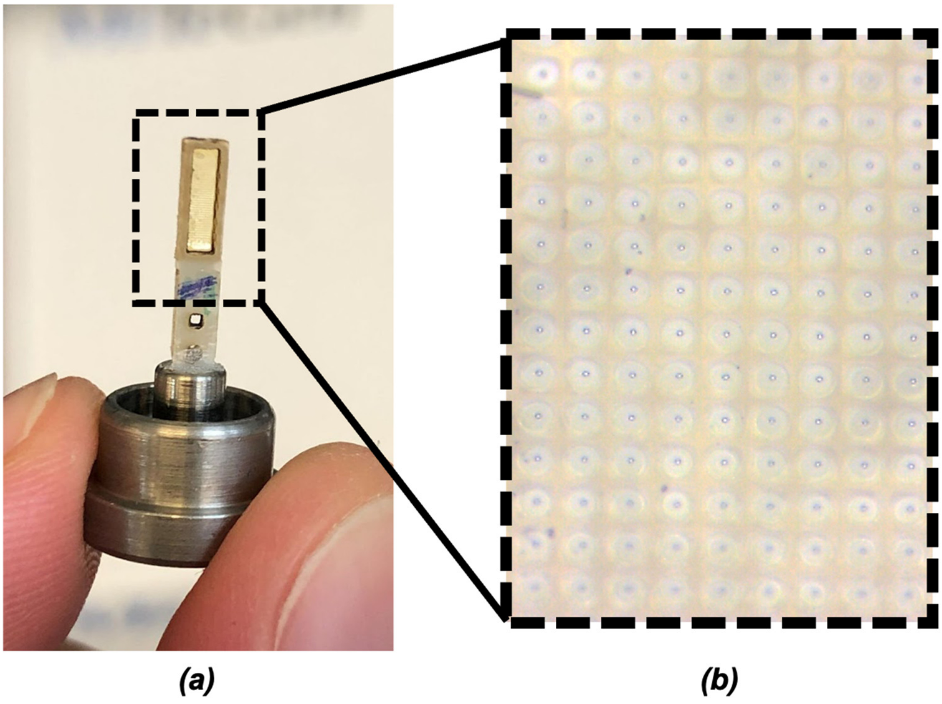

Figure S1 Serial sample support chip used for RT SSX of PTP1B. *(a)* Photograph of an example chip with its goniometer-compatible base. *(b)* Micrograph of crystal-loading facets on the face of a chip.

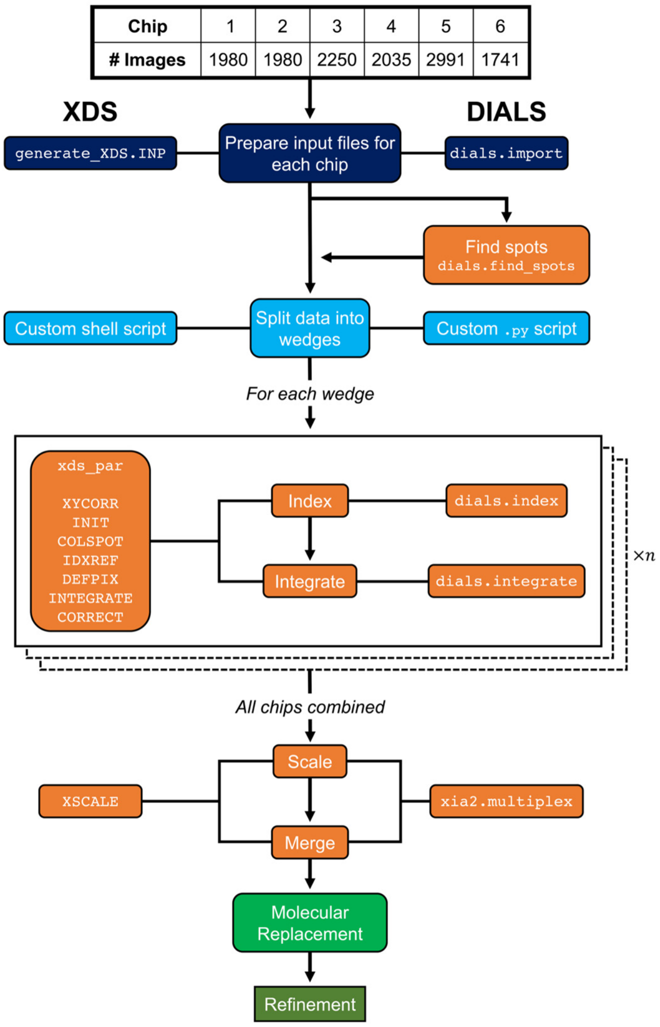

Figure S2 Flowchart of data reduction workflow. Two pipelines, XDS and DIALS, were used in parallel to process our SSX data. Both involved similar workflows (central flow), albeit with slight variations (left vs. right). n refers to the number of wedges.

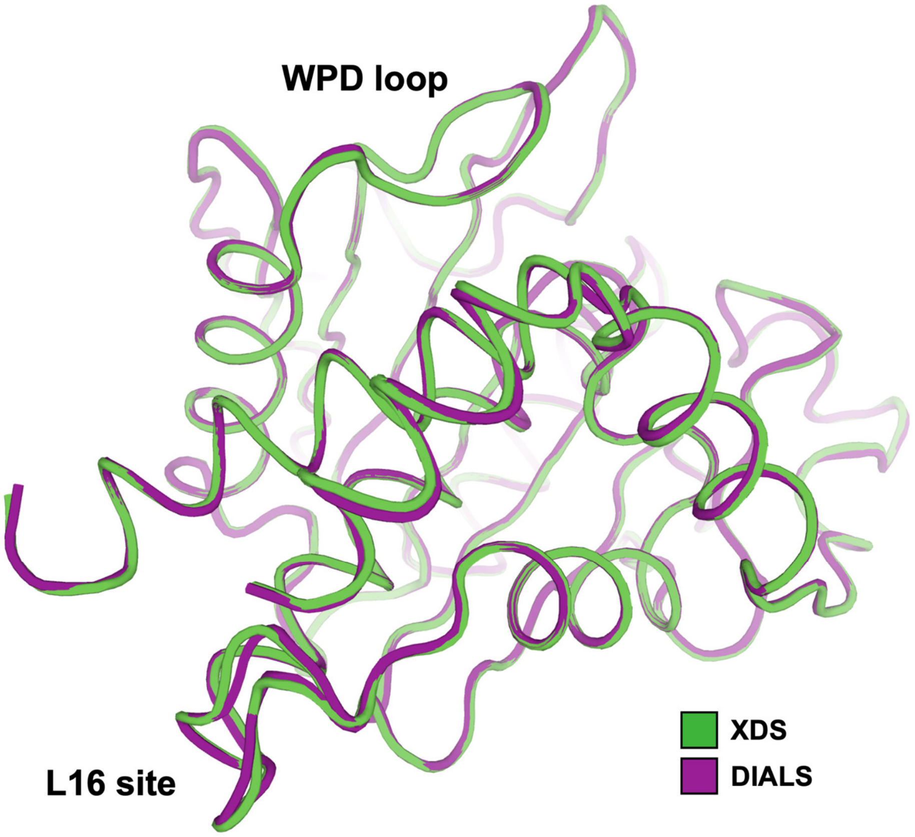

Figure S3 Cα alignment of final models from XDS and DIALS data processing pipelines. The Cα RMSD between XDS and DIALS processed models is 0.21 Å.

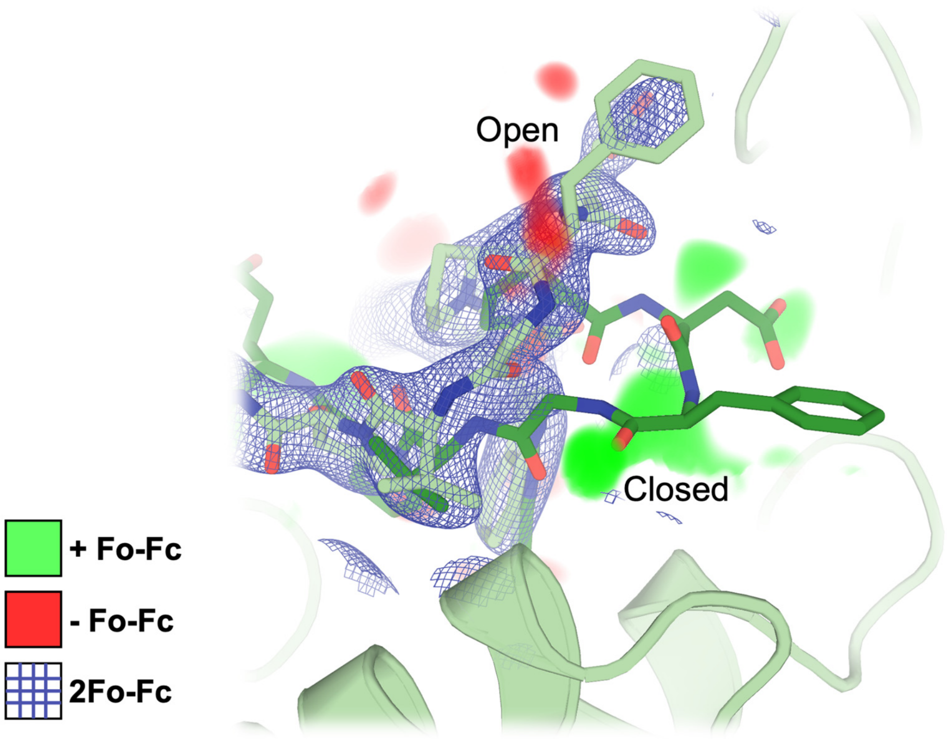

Figure S4 A dual-conformation model of the WPD loop lacks electron density support. 2Fo-Fc density at 1 σ and Fo-Fc density at +/- 3.0 σ.

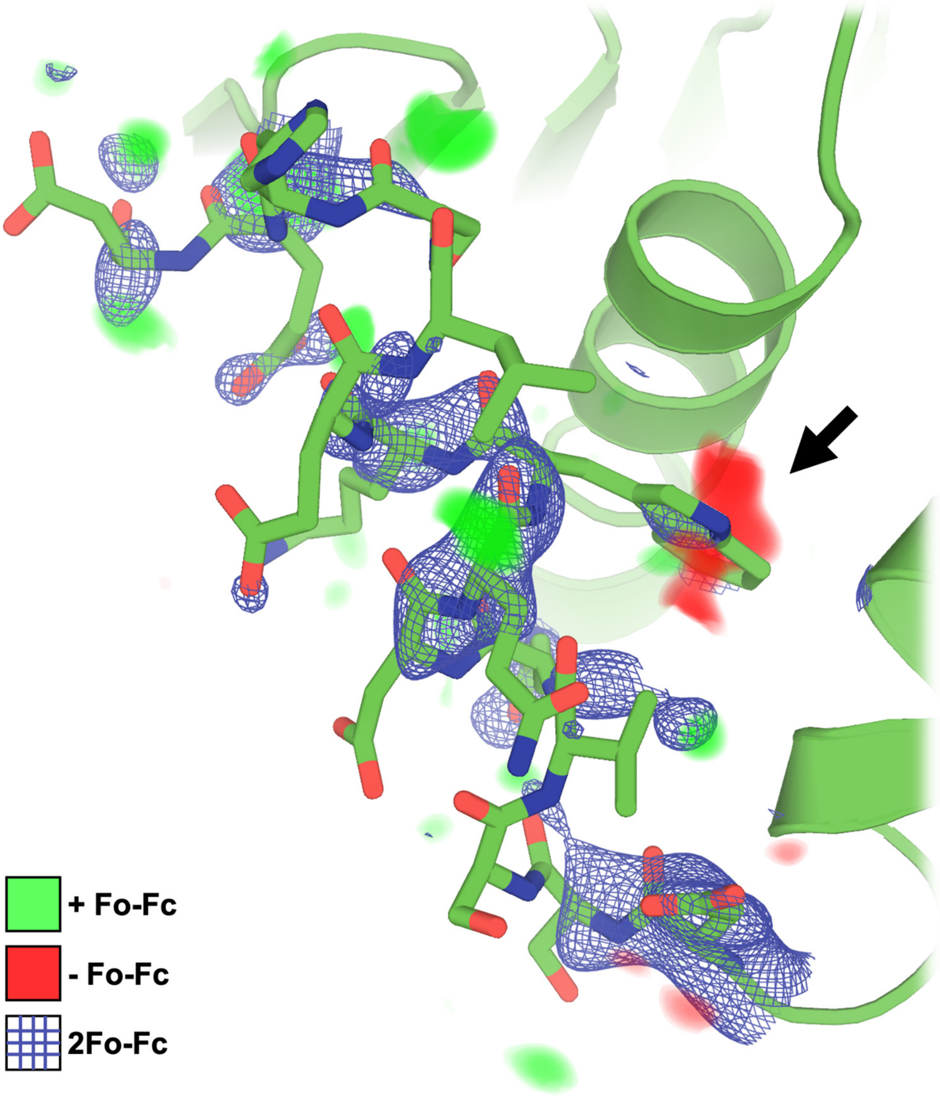

Figure S5 A model with the ordered α7 helix modeled at partial occupancy has only minimal electron density support. 2Fo-Fc density at 1 σ and Fo-Fc density at +/- 3.0 σ. Note the negative Fo-Fc density peak for the Trp291 "anchor" when it is modeled as occupying the allosteric BB site (arrow).

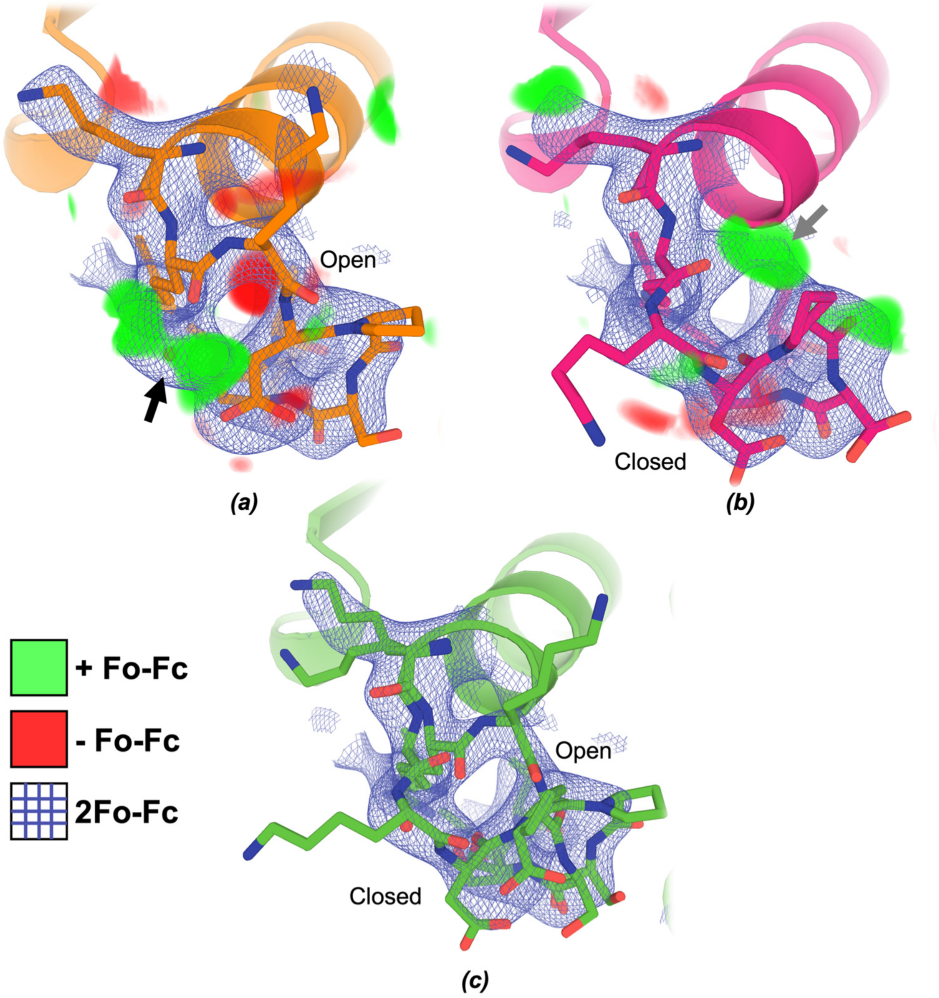

Figure S6 Omit maps confirm that Loop 16 has alternate conformations. 2Fo-Fc (1 σ) and Fo-Fc (+/- 3 σ) maps are shown for models with individual states of Loop 16 in the L16 site omitted. *(a)* Closed state omitted. Positive Fo-Fc density (green) is apparent despite the presence of a modeled water in the 2Fo-Fc map (black arrow), indicating further model building is required. *(b)* Open state omitted. Positive Fo-Fc density is present for the open L16 conformation (gray arrow), further indicating the model should be built sampling both the open and closed L16 states. *(c)* Final model including both states as alternate conformations (same as **Fig. 2d**).
